# Integrative characterization of intraductal tubulopapillary neoplasm (ITPN) of the pancreas and associated invasive adenocarcinoma

**DOI:** 10.1038/s41379-022-01143-2

**Published:** 2022-09-02

**Authors:** Andrea Mafficini, Michele Simbolo, Tatsuhiro Shibata, Seung-Mo Hong, Antonio Pea, Lodewijk A. Brosens, Liang Cheng, Davide Antonello, Concetta Sciammarella, Cinzia Cantù, Paola Mattiolo, Sergio V. Taormina, Giuseppe Malleo, Giovanni Marchegiani, Elisabetta Sereni, Vincenzo Corbo, Gaetano Paolino, Chiara Ciaparrone, Nobuyoshi Hiraoka, Daniel Pallaoro, Casper Jansen, Michele Milella, Roberto Salvia, Rita T. Lawlor, Volkan Adsay, Aldo Scarpa, Claudio Luchini

**Affiliations:** 1 Department of Diagnostics and Public Health, Section of Pathology, University and Hospital Trust of Verona, Verona, Italy; 2 ARC-Net Research Center, University of Verona, Verona, Italy; 3 Division of Cancer Genomics, National Cancer Center Research Institute, and Laboratory of Molecular Medicine, The Institute of Medical Sciences, The University of Tokyo, Tokyo, Japan; 4 Department of Pathology, Asan Medical Center, University of Ulsan College of Medicine, Seoul, South Korea; 5 Department of General and Pancreatic Surgery - The Pancreas Institute, University and Hospital Trust of Verona, Verona, Italy; 6 Department of Pathology, University Medical Center, Utrecht, The Netherlands; 7 Department of Pathology and Laboratory Medicine, Warren Alpert Medical School of Brown University and Lifespan Academic Medical Center, Providence, RI USA; 8 Division of Pathology and Clinical Laboratories, National Cancer Center Hospital, Tokyo, Japan; 9 Laboratory for Pathology Eastern Nertherlands, Hengelo, The Netherlands; 10 Department of Medicine, Section of Oncology, University and Hospital Trust of Verona, Verona, Italy; 11 Department of Pathology, Koç University Hospital and Koç University Research Center for Translational Medicine (KUTTAM), Istanbul, Turkey

## Abstract

Pancreatic intraductal tubulopapillary neoplasm (ITPN) is a recently recognized intraductal neoplasm. This study aimed to clarify the clinicopathologic and molecular features of this entity, based on a multi-institutional cohort of 16 pancreatic ITPNs and associated adenocarcinomas. The genomic profiles were analyzed using histology-driven multi-regional sequencing to provide insight on tumor heterogeneity and evolution. Furthermore, an exploratory transcriptomic characterization was performed on eight invasive adenocarcinomas. The clinicopathologic parameters and molecular alterations were further analyzed based on survival indices. The main findings were as follows: 1) the concomitant adenocarcinomas, present in 75% of cases, were always molecularly associated with the intraductal components. These data definitively establish ITPN as origin of invasive pancreatic adenocarcinoma; 2) alterations restricted to infiltrative components included mutations in chromatin remodeling genes *ARID2*, *ASXL1*, and *PBRM1*, and *ERBB2*-*P3H4* fusion; 3) pancreatic ITPN can arise in the context of genetic syndromes, such as *BRCA*-germline and Peutz–Jeghers syndrome; 4) mutational profile: mutations in the classical PDAC drivers are present, but less frequently, in pancreatic ITPN; 5) novel genomic alterations were observed, including amplification of the Cyclin and *NOTCH* family genes and *ERBB2*, fusions involving *RET* and *ERBB2*, and *RB1* disruptive variation; 6) chromosomal alterations: the most common was 1q gain (75% of cases); 7) by transcriptome analysis, ITPN-associated adenocarcinomas clustered into three subtypes that correlate with the activation of signaling mechanism pathways and tumor microenvironment, displaying squamous features in their majority; and 8) *TP53* mutational status is a marker for adverse prognosis. ITPNs are precursor lesions of pancreatic cancer with a high malignant transformation risk. A personalized approach for patients with ITPN should recognize that such neoplasms could arise in the context of genetic syndromes. *BRCA* alterations, *ERBB2* and *RET* fusions, and *ERBB2* amplification are novel targets in precision oncology. The *TP53* mutation status can be used as a prognostic biomarker.

## Introduction

Pancreatic intraductal tubulopapillary neoplasm (ITPN) is recognized as a subtype of pancreatic neoplasms that form a heterogeneous group of intraductal lesions, which also includes intraductal papillary mucinous neoplasm (IPMN) and intraductal oncocytic papillary neoplasm (IOPN)^1^. ITPN accounts for up to 3–5% of all intraductal pancreatic neoplasms^1–4^.

Similar to IPMN, ITPN shows various intraductal growth degrees. However, compared to IPMN, ITPN is less frequently cystic, forming instead fleshy and solid masses in the involved ducts^4,5^. Histologically, ITPNs are hypercellular tumors comprising nodules of back-to-back tubular glands with absent or very scant mucin formation^1,3,6–8^. The tubular areas are predominant, whereas papillary components are limited. In addition to architectural complexity, ITPN displays uniform high-grade cytological atypia with numerous mitotic figures and frequent foci of necrosis. Intra-cytoplasmic and extra-cellular mucins are consistently absent^4,6–8^.

Pancreatic ITPN is a presumed precursor of invasive ductal adenocarcinoma, although definitive evidence is still lacking. Concomitant adenocarcinomas have been reported in up to 70% of cases at diagnosis^8^. Despite the high-grade cytological and architectural features and the frequent association with concomitant invasive cancer, ITPN usually has a more favorable prognosis than conventional pancreatic ductal adenocarcinoma (PDAC), even when associated infiltrative lesions are present. However, a small subset of patients presents with locally advanced or metastatic disease at diagnosis or will develop local recurrence or distant metastases after surgical resection; thus, better comprehension of this lesion type is warranted.

ITPN has a distinct mucin immunohistochemical profile, rendering immunohistochemistry (IHC) an important supportive tool in the ITPN diagnosis. ITPNs are usually characterized by the expression of MUC1 and MUC6 and generally lack expression of the MUC5AC and MUC2 proteins^1,7^. Moreover, pancreatic ITPN is molecularly distinct from IPMN and conventional ductal adenocarcinoma, showing rare (but not absent) mutations in the *KRAS* and *TP53* genes and more common *PI3KCA* mutations and *FGFR2* fusions^9–13^.

In the present study, we performed a multi-institutional analysis of the molecular profile of different ITPN components (tubular and papillary areas) and concomitant invasive cancers through histology-driven multi-regional sequencing. This study aimed to clarify the genomic features of pancreatic ITPN, including tumor heterogeneity and the molecular progression to invasive cancers. Based on the results of our analyses, we provide specific insights into molecular markers with clinical impact and suggest possible novel targets for precision oncology.

## Materials and methods

### Case selection and clinicopathologic analysis

The following electronic databases were searched for pancreatic ITPN cases: Verona University and Hospital Trust (Verona, Italy), National Cancer Center Research Institute (Tokyo, Japan), Asan Medical Center (Seoul, South Korea), University Medical Center (Utrecht, The Netherlands), and Indiana University (Indianapolis, IN, USA). Cases with material available for molecular analysis were selected. Our cohort comprised 16 cases, which were subsequently confirmed by histology performed by two pancreatic pathologists. All cases were negative for BCL10, chromogranin A, and synaptophysin. Medical records and electronic databases were used to obtain supplementary clinicopathologic data, including prognostic outcomes. Cases were staged using the American Joint Committee on Cancer staging, 8^th^ edition^14^.

### Multi-regional massive parallel DNA sequencing

To understand better tumor heterogeneity and evolution, a multi-regional sequencing approach for genomic analysis was adopted. The most representative inclusion from each case were selected for analysis. The tubular area and the papillary region for the 16 ITPNs were then selected. Co-occurring adenocarcinomas, when present, were also analyzed. Genomic DNA was obtained from formalin-fixed, paraffin-embedded tissues after enrichment for neoplastic cellularity, using manual microdissection. DNA was extracted and quantified as previously described^15^, using the GeneRead DNA FFPE kit (Qiagen - Hilden, Germany) according to the manufacturer’s instructions.

DNA sequencing was performed for both tubular and papillary tumor components, following the previously described SureSelectXT HS CD Glasgow Cancer Core assay (www.agilent.com), hereafter referred to as CORE^16,17^. The CORE panel spans 1.8 Mb of the genome and searches 174 genes for somatic mutations, copy number alterations, and structural rearrangements. The details of the targeted genes are reported in Supplementary Table 1. Sequencing was performed on a NextSeq 500 (Illumina, San Diego, CA, USA) loaded with two captured library pools using a high-output flow cell and 2 × 75 bp paired-end sequencing.

CORE panel analysis started with demultiplexing performed with FASTQ Generation v1.0.0 on the BaseSpace Sequence Hub (https://basespace.illumina.com, last access 11/16/2021). Forward and reverse reads from each demultiplexed sample were aligned to the human reference genome (version hg38/GRCh38) using Burrows-Wheeler Aligner version 0.7.17-r1188^18^. Mapped reads were subjected to PCR duplication removal and indexed, using biobambam2 v2.0.146 (https://gitlab.com/german.tischler/biobambam2.git; last access 11/16/2021)^19^. Coverage statistics were calculated using the same software^20^. Single nucleotide variants were identified using shearwater^21^. Small (<200 bp) insertions and deletions were identified using Pindel version 0.2.5b8^22^. All candidate mutations were manually reviewed using the Integrative Genomics Viewer version 2.4 to exclude sequencing artifacts^23^.

Microsatellite instability was calculated using the method described by Papke et al.^24^. Copy number alterations of targeted genes were detected using the GeneCN software (https://github.com/wwcrc/geneCN; last access 06/30/2021). Structural rearrangements were detected using the BRASS software^25^, and visually reviewed using the Integrative Genomics Viewer, version 2.4^23^.

Tumor variants were classified as benign (class 1), likely benign (class 2), variant of uncertain significance (class 3), likely pathogenic (class 4), or pathogenic (class 5), according to the guidelines of the American College of Medical Genetics and Genomics and the Association for Molecular Pathology^26^.

### Transcriptome analysis

Gene-expression analysis of 20,815 human genes was performed on the co-occurring adenocarcinomas to obtain their transcriptomic profile, according to previously described methods^27^. Briefly, libraries were prepared using the Ampliseq Transcriptome Human Gene Expression Kit (Thermo Fisher Scientific, Waltham, MA, USA) with 1 µg of retrotranscribed RNA for each multiplex PCR amplification. The AmpliSeqRNA plugin generated each sample’s expression data (counts per transcript). Counts were normalized and transformed using the DESeq2 package for R^28^. Visualization and clustering were performed using the ComplexHeatmap package for R^29^. The NbClust package was adopted to estimate the best number of clusters. Then, a hybrid hierarchical k-means approach was used to perform principal component analysis and to design a dendrogram showing the relationships between samples. To verify the resulting associations between samples, unsupervised consensus clustering was performed using ConsensusClusterPlus. For tumor classification, pancreatic cancer signatures were retrieved from studies performed by Bailey et al.^30^, Collisson et al.^31^, and Moffitt et al.^32^, and cluster-specific enriched gene sets were determined using the normalized count matrix. We applied gene set enrichment analysis (GSEA) using the GAGE-R package between clusters to obtain significant pairwise up- and down-regulated pathways^33^. We performed z-score normalization of pathway scores in each cluster.

### Chromogenic multiplex IHC and additional IHC

Adenocarcinoma gene expression profiling related to immune microenvironment composition was cross-validated using chromogenic multiplex IHC analysis as previously described^27^. Based on the results of the transcriptome analysis, two T-lymphocyte markers, CD4 (labeled in red) and CD8 (DAB), and the class 2 macrophage marker CD163 (green) were selected for this study. Cells were considered “positive” when the cell membrane was stained. The expression of these markers was evaluated as previously reported, using a semi-quantitative (0–5) scoring system: 0 = negative (no stained cells), 1 = rare (1–10 positive cells per high-power field, HPF; 400× magnification), 2 = low (11–20 positive cells per HPF), 3 = moderate (21–30 positive cells per HPF), 4 = high (31–50 positive cells per HPF), and 5 = very high (>50 positive cells per HPF)^27^.

In the case of *ERBB2* amplification, a specific IHC analysis for Her2 (Hercep test, Dako, Germany) was performed. Finally, all cases were tested for p53 with IHC (clone: DO-7, 1:50 dilution, Novocastra, UK).

### Survival analysis

Univariate and multivariate Cox regression analyses were performed to investigate any association between clinicopathologic and molecular data, and survival outcomes. The outcomes considered were overall survival, cancer-specific survival, disease-free survival, and composite outcome. Multivariable analysis was planned using the factors significantly associated with the survival outcomes of interest with a *p*-value < 0.10 in the univariate analyses. Data from the Cox regression analyses were graphically reported using Kaplan–Meier curves. The results were presented as hazard ratios with a 95% confidence interval. Statistical analyses were performed using SPSS version 20.0 (Chicago, IL, USA).

## Results

### Clinicopathologic analysis

The crucial clinicopathological features of the 16 cases are summarized in Table 1. Five patients were men (31.2%) and 11 were women (68.8%), with an average age at diagnosis of 63.2 years (range 47–76). Three cases (18.8%) were incidentally diagnosed in asymptomatic individuals; of these, two were diagnosed during routine follow-up for genetic syndromes, such as hereditary breast and ovarian cancer syndrome (HBOC) and Peutz–Jeghers syndrome.

**Table 1:** Summary of the most important clinicopathologic data of pancreatic ITPNs and concomitant invasive adenocarcinomas.

N case	Sex, age	Site in the pancreas	Associated cancer	Size of the whole lesion (invasive component)	pTNM (*T* = size of the invasive component)	Tumor stage	VI	PNI	R	Involved ducts	Relevant clinical history and symptoms	Main radiologic findings	Survival (months)
1	M, 76	Head-body-tail	Yes	100 mm (25 mm)	pT2N1M0	IIB	Yes	No	R0	Main + branch	Abdominal pain	Solid-cystic lesion	AF (65)
2	F, 71	Head	No	20 mm	pTisN0M0	0	n/a	n/a	R0	Main	Incidental finding	Solid-cystic lesion	AF (62)
3	M, 68	Head	Yes	50 mm (36 mm)	pT2N1M0	IIB	Yes	No	R0	Main + branch	Jaundice	Solid-cystic lesion	AF (31)
4	F, 75	Head, body, tail	Yes	130 mm (60 mm)	pT3N0M0	IIA	no	No	R1	Main + branch	Abdominal pain	Solid-cystic lesion	DO (0)
5	M, 72	Head	No	12 mm	pTisN0M0	0	n/a	n/a	R0	Main	Jaundice	Solid-cystic lesion	AF (15)
6	F, 50	Head-body-tail	No	36 mm	pTisN0M0	0	n/a	n/a	R0	Main + branch	Epigastric pain, steatorrhea, weight loss	Enlargement of the pancreatic gland, Solid-cystic	AF (54)
7a^a^	M, 57	Tail	No	16 mm	pTisN0M0	0	n/a	n/a	R0	Main	Acute pancreatitis	Cystic lesion	AD (38)
7b^a^	M, 59	Head-body	Yes	25 mm (12 mm)	pT1cN0M0	IA	Yes	Yes	R0	Main + branch	Diagnosed on follow-up for ITPN	Solid-cystic	AF (8)
8	F, 64	Body-tail	Yes	70 mm (23 mm)	pT2N1M0	IIB	Yes	Yes	R0	Main + branch	Abdominal pain	Solid-cystic	AF (9)
9	M, 60	Body-tail	Yes	60 mm (31 mm)	pT2N0M0	IB	Yes	Yes	R0	Main + branch	Peutz–Jeghers syndrome; Diagnosed on follow-up	Solid-cystic	AF (27)
10	F, 47	Body	Yes	25 mm (3 mm)	pT1aN0M0	IA	No	No	R0	Main	n/a	Solid lesion	AF (3)
11	F, 63	Head	Yes	35 mm (22 mm)	pT2N2M0	III	Yes	Yes	R0	Main	Jaundice, pruritus	Solid lesion	AD (54); liver metastasis (9)
12	F, 60	Body-tail	Yes	90 mm (45 mm)	pT3N2M0	III	Yes	Yes	R0	Main	Fatigue, weight loss, fever, abdominal pain	Solid lesion	DD (2); recurrence (1)
13	F, 72	Head	Yes	25 mm (7 mm)	pT1bN2M0	III	Yes	Yes	R0	Main	Epigastric pain, weight loss, anorexia	Solid lesion	n/a
14	F, 59	Head	Yes	25 mm (9 mm)	pT1bN0M0	IA	Yes	Yes	R0	Main	Previous ovarian serous carcinoma	Solid lesion	DO (21)
15	F, 64	Head-body-tail	Yes	66 mm (4 mm)	pT1aN0M0	IA	Yes	Yes	R0	Main + branch	Abdominal pain	Solid-cystic	DO (13)
16	F, 54	Body-tail	Yes	35 mm (30 mm)	pT2N2M1	IV	Yes	Yes	R1	n/a	Dyspepsia, abdominal pain	Solid-cystic	AF (4)

*F* female, *M* male, *pTNM* pathologic TNM staging, *VI* vascular invasion, *PNI* perineural invasion, *R* surgical margin status (R0 negative; R1 positive), *AF* alive free of disease, *AD* alive with disease, *DO* dead of other causes, *DD* death of disease, *ITPN* intraductal tubulopapillary neoplasm, *n/a* not available.

^a^Patient n. 7a experienced a tumor relapse (7b); therefore, both cases (7a and 7b) are reported.

At diagnosis, co-occurring invasive adenocarcinoma was present in 12 cases (75%), represented by glandular/tubular adenocarcinoma. Regarding tumor stage, four cases (25%) were resected at stage 0 (i.e., non-invasive), four (25%) at stage I, four (25%) at stage II, three (18.8%) at stage III, and one case (6.2%) at stage IV due to the presence of a single liver metastasis.

Follow-up data were available for 15/16 patients. The majority (10, 62.5%) were alive and disease-free at the last follow-up (average follow-up time: 27.9 months). Two pancreatic lesions were analyzed in one patient; an initial lesion during surgical resection for a non-invasive ITPN (case #7a), and a later lesion during local adenocarcinoma tumor relapse (case #7b), observed 38 months after the surgical resection. After surgical re-intervention for relapse, the patient remains alive and disease-free at the most recent follow-up (8 months after re-intervention).

### Molecular analysis

#### Multiregional massive parallel sequencing

**Fig. 1:**
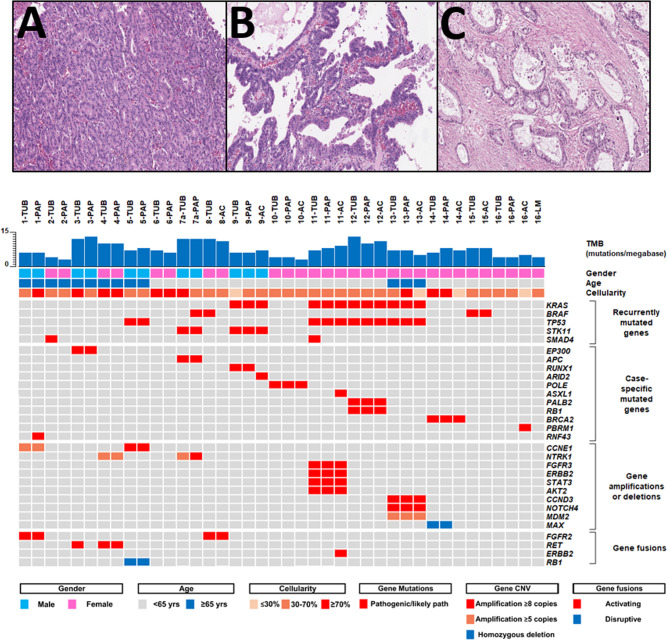
Summarizing figure of histology-based genomic analysis. Histological images of the tumor areas selected for multi-regional sequencing: (**A**) tubular component; (**B**) papillary component; (**C**) adenocarcinoma. Hematoxylin-eosin staining at 10× magnification for observation of structures. The graphs show the results of the genomic analysis of all patients at diagnosis, represented per tumor component. Each case is identified with a number followed by an acronym, indicating the specific tumor region (TUB tubular, PAP papillary, AC adenocarcinoma).

All cases were investigated using multi-regional sequencing to assess their genomic profiles. In the single metastatic case, we investigated the invasive pancreatic adenocarcinoma and the liver metastasis in addition to the papillary and tubular intraductal components. Thus, we provided the molecular characterization of tubular and of papillary intraductal components of six cases, whereas in the remaining 10 cases, the co-occurring adenocarcinoma was also investigated (Fig. 1). For two cases (#1 and #3), the invasive component was not suitable for molecular analysis. The mutational profiles and copy number variations are summarized in Table 2 and structural alterations are shown in Table 3.

**Table 2:** Pathogenic/likely pathogenic mutations and gene copy-number variations identified in pancreatic ITPN and associated invasive carcinoma.

ID case	Histology	TMB	MSI	Clinically relevant SNV					CNV		
				Gene	Variation	Mutation type	Freq (%)	Class	Gene	Variation	# of copies
1	Tubular	5.6	MSS	none					*FGFR1*	Gain	3.0
									*RAD50*	LOH	1.0
	Papillary	5.6	MSS	*RNF43*	c.571+1G>A	Substitution – splice site	13	4	*FGFR1*	Gain	3.4
									*RAD50*	LOH	1.0
2	Tubular	3.9	MSS	*SMAD4*	p.D415fs*20	Deletion – frameshift	27	5	*NOTCH1*	Gain	3.6
									*CCNE1*	Ampl	5.0
	Papillary	2.8	MSS	none					*FGFR4*	Gain	3.5
									*CCNE1*	Ampl	5.5
3	Tubular	11.7	MSS	*EP300*	c.3591-1G>A	Splice site variation	48	4	none		
	Papillary	13.9	MSS	*EP300*	c.3591-1G>A	Splice site variation	25	4	none		
4	Tubular	10.0	MSS	none					*NTRK1*	Ampl	5.0
	Papillary	10.0	MSS	none					*NTRK1*	Ampl	5.0
5	Tubular	7.8	MSS	*TP53*	p.Y220C	Substitution – missense	48	5	*TP53*	LOH	1.0
									*NOTCH3*	Ampl	5.0
									*CCNE1*	Ampl	29.0
	Papillary	7.2	MSS	*TP53*	p.Y220C	Substitution – missense	24	5	*TP53*	LOH	1.0
									*NOTCH3*	Gain	4.0
									*CCNE1*	Ampl	29.0
6	Tubular	6.6	MSS	none					None		
	Papillary	6.1	MSS	none					None		
7a^a^	Tubular	11.7	MSS	*BRAF*	p.V600E	Substitution – missense	35	5	*NTRK1*	Ampl	6.0
				*APC*	p.E1544*	Substitution – nonsense	5	5	*STK11*	LOH	1.0
				*STK11*	p.Q159*	Substitution – nonsense	51	5			
	Papillary	11.7	MSS	*BRAF*	p.V600E	Substitution – missense	35	5	*NTRK1*	Ampl	8.0
				*APC*	p.E1544*	Substitution – nonsense	45	5	*STK11*	LOH	1.0
				*STK11*	p.Q159*	Substitution – nonsense	47	5			
7b^a^	AC	11.4	MSS	*BRAF*	p.V600E	Substitution – missense	21	5	*NTRK1*	Ampl	13.1
				*STK11*	p.Q159*	Substitution – nonsense	20	5	*STK11*	LOH	0.9
8	Tubular	11.6	MSS	none					*MDM2*	Gain	4.0
	AC	11.0	MSS	none					*MDM2*	Gain	4.0
9	Tubular	5.6	MSS	*KRAS*	p.G12S	Substitution – missense	11	5	*STK11*	LOH + gain	3.1
				*RUNX1*	p.S318Ffs*282	Deletion – frameshift	12	5			
				*STK11*	c.921-1G>C	Substitution – splice site	68	5			
	Papillary	6.1	MSS	*KRAS*	p.G12S	Substitution – missense	14	5	*STK11*	LOH + gain	3.0
				*RUNX1*	p.S318Ffs*282	Deletion – frameshift	3	5			
				*STK11*	c.921-1G>C	Substitution – splice site	57	5			
	AC	6.7	MSS	*KRAS*	p.G12S	Substitution – missense	15	5	*STK11*	LOH + gain	3.0
				*STK11*	c.921-1G>C	Substitution – splice site	66	5			
				*ARID2*	p.S1476Cfs*26	Deletion – frameshift	3	4			
10	Tubular	3.9	MSS	*POLE*	c.4149+2dupT	Insertion – splice site	40	4			
	Papillary	3.9	MSS	*POLE*	c.4149+2dupT	Insertion – splice site	42	4			
	AC	3.3	MSS	*POLE*	c.4149+2dupT	Insertion – splice site	44	4			
11	Tubular	7.2	MSS	*KRAS*	p.G12D	Substitution – missense	34	5	*FGFR3*	Ampl	6.5
				*TP53*	p.R248W	Substitution – missense	55	5	*APC*	LOH	1.0
				*SMAD4*	p.R445*	Substitution – nonsense	55	5	*ERBB2*	Ampl	10.3
									*STAT3*	Ampl	8.2
									*AKT2*	Ampl	14.6
	Papillary	8.3	MSS	*KRAS*	p.G12D	Substitution – missense	35	5	*FGFR3*	Ampl	6.5
				*TP53*	p.R248W	Substitution – missense	65	5	*APC*	LOH	1.0
									*ERBB2*	Ampl	9.6
									*STAT3*	Ampl	7.3
									*AKT2*	Ampl	9.9
	AC	9.4	MSS	*KRAS*	p.G12D	Substitution – missense	41	5	*FGFR3*	Gain	4.0
				*TP53*	p.R248W	Substitution – missense	47	5	*APC*	LOH	1.0
				*ASXL1*	p.Q1074*	Substitution – nonsense	5	4	*ERBB2*	Ampl	8.6
									*STAT3*	Ampl	6.5
									*AKT2*	Ampl	7.6
12	Tubular	12.7	MSS	*KRAS*	p.G12V	Substitution – missense	24	5	None		
				*TP53*	p.L194H	Substitution – missense	59	5			
				*PALB2*	p.E13K	Substitution – missense	22	4			
				*RB1*	c.138-1G>T	Substitution – splice site	70	4			
	Papillary	10.1	MSS	*KRAS*	p.G12V	Substitution – missense	11	5	*NOTCH1*	Gain	4.0
				*TP53*	p.L194H	Substitution – missense	60	5			
				*PALB2*	p.E13K	Substitution – missense	21	4			
				*RB1*	c.138-1G>T	Substitution – splice site	45	4			
	AC	11.1	MSS	*KRAS*	p.G12V	Substitution – missense	20	5	None		
				*TP53*	p.L194H	Substitution – missense	35	5			
				*PALB2*	p.E13K	Substitution – missense	28	4			
				*RB1*	c.138-1G>T	Substitution – splice site	52	4			
13	Tubular	6.6	MSS	*KRAS*	p.G12D	Substitution – missense	34	5	*CCND3*	Ampl	9.0
				*TP53*	p.Y220C	Substitution – missense	35	5	*NOTCH4*	Ampl	9.0
									*MDM2*	Ampl	5.0
	Papillary	6.6	MSS	*KRAS*	p.G12D	Substitution – missense	44	5	*CCND3*	Ampl	9.0
				*TP53*	p.Y220C	Substitution – missense	42	5	*NOTCH4*	Ampl	9.0
									*MDM2*	Ampl	5.0
	AC	5	MSS	*KRAS*	p.G12D	Substitution – missense	10	5	*CCND3*	Ampl	9.0
				*TP53*	p.Y220C	Substitution – missense	10	5	*NOTCH4*	Ampl	9.0
									*MDM2*	Ampl	5.0
14	Tubular	6.5	MSS	*BRCA2*	p.Q2960*	Stop gain	85	5	*MAX*	Hom del	0.0
	Papillary	8.1	MSS	*BRCA2*	p.Q2960*	Stop gain	93	5	*MAX*	Hom del	0.0
	AC	6.7	MSS	*BRCA2*	p.Q2960*	Stop gain	54	5	NA^b^		
15	Tubular	8.3	MSS	*BRAF*	p.V600E	Substitution – missense	32	5	*STK11*	LOH	1.0
									*NOTCH3*	LOH	1.0
									*JAK3*	Gain	3.0
	AC	7.8	MSS	*BRAF*	p.V600E	Substitution – missense	32	5	*STK11*	LOH	1.0
									*NOTCH3*	LOH	1.0
									*JAK3*	Gain	3.0
16	Tubular	4.3	MSS	none					None		
	Papillary	4.3	MSS	none					None		
	AC	5.4	MSS	*PBRM1*	p.D554fs*4	Deletion – frameshift	13	4	None		
	Liver met.	4.3	MSS	none					None		

*TMB* tumor mutational burden, *MSI* microsatellite instability, *MSS* microsatellite stable, *SNV* single nucleotide variants, *CNV* copy number variations, *AC* adenocarcinoma, *N/A* not available, *LOH* loss of heterozygosity (1 copy), *Gain* >2 copies, *Ampl* amplification (>4 copies), *met* metastasis, *Class* clinical Impact class according to AMGP/AMP guidelines (5: pathogenic; 4: likely pathogenic; Richards et al. Genet Med 2015).

^a^7a pancreatic resection for ITPN; 7b: local relapse as adenocarcinoma.

^b^Neoplastic cellularity was too low for CNV analysis.

**Table 3:** Structural alterations of pancreatic ITPN and associated invasive carcinoma.

ID case	Histology	Involved genes		Type of alteration
		Gene 1 (region)	Gene 2 (region)	
1	Tubular	*FGFR2* (exon 17)	*HSD17B4* (exon 13)	Fusion
	Papillary	*FGFR2* (exon 17)	*HSD17B4* (exon 13)	Fusion
3	Tubular	*RET* (exon 12)	*C14orf93* (exon 3)	Fusion
	Papillary	–	–	–
4	Tubular	*TRIM24* (exon 9)	*RET* (exon 12)	Fusion
	Papillary	*TRIM24* (exon 9)	*RET* (exon 12)	Fusion
5	Tubular	*LMNA*	*RB1*	Translocation^a^
	Papillary	*LMNA*	*RB1*	Translocation^a^
8	Tubular	*FGFR2* (exon 17)	*SYCP1* (exon 24)	Fusion
	AC	*FGFR2* (exon 17)	*SYCP1* (exon 24)	Fusion
11	Tubular	–	–	–
	Papillary	–	–	–
	AC	*ERBB2* (exon 24)	*P3H4* (exon 2)	Fusion

*ITPN* intraductal tubulopapillary neoplasm.

^a^This is a translocation by asymmetric breakdown and repair of chromosomes 1 (1q22, *LMNA* gene) and 13 (13q14.2, *RB1* gene), resulting in a dicentric and an acentric chromosome that contains the distal parts of the q arms, welded together. In particular, regarding *LMNA* and *RB1*, there is the 3’-3 ‘and 5’-5’ junction of the truncates, resulting in the loss of genes.

Sequencing revealed recurrent mutations in the classical PDAC drivers: *KRAS* mutations in four cases (25%), in both the ITPN and the concomitant infiltrating adenocarcinoma; *TP53* mutations in four cases (25%), three of which had a co-occurring adenocarcinoma; *SMAD4* mutations in two cases (12.5%), restricted to the tubular area and not altered in either the papillary or the adenocarcinoma, the latter present in only one case; *BRAF* was mutated in two cases (12.5%), both displaying the same V600E mutation; *RNF43* was mutated only in the papillary component of a noninvasive case; and no mutations were detected in *CDKN2A* or *GNAS* in any of the cases. None of the ITPN samples showed microsatellite instability. The four cases harboring *TP53* mutations showed aberrant staining pattern in p53 IHC, with strong and diffuse nuclear positivity of >90% tumor cells (Supplementary Fig. 1). Other cases were interpreted as wild type.

Two ITPNs associated with concomitant adenocarcinoma (case #9 and #14) were detected as part of the spectrum of familial cancer syndromes. One case was diagnosed in a patient with Peutz–Jeghers syndrome; the germline variation was associated with LOH of *STK11*. The other case was detected in a patient with HBOC syndrome carrying a *BRCA2* germline variation coupled with LOH on chromosome 13.

Copy number variations of the Cyclin family genes were noted in three cases (18.75% of cases), in particular, *CCNE1* was amplified in two cases (12.5%) and *CCND3* in one case (6.25%). Gene gain/amplification is frequently observed in the *NOTCH* and *FGFR* families in ITPN. Here, alterations in *FGFR* involved two cases with gene gain (12.5%) and one with amplification (6.25%), and in *NOTCH*, two cases with gain (12.5%) and two with amplification (12.5%). We also observed *NTRK1* amplification in all the components in two cases (12.5%); one was the relapsing case and the alteration was maintained in the recurrent neoplasm. Finally, *ERBB2* was amplified in all the components of one ITPN sample with concomitant adenocarcinoma. In IHC staining, Her2 expression showed a heterogeneous pattern, from a weak to a focally strong positivity (although there are no specific guidelines for assessing Her2 in pancreatic tumors; Supplementary Fig. 2).

Six cases harbored structural genomic alterations (Table 3). Among these, five showed gene fusions and one showed translocation. *RET* was fused with *C14orf93* or *TRIM24*, *FGFR2* with *STCP1* or *HSD17B4*, and *ERBB2* with *P3H4*. Translocation by asymmetric breakdown and repair of chromosome 1 in *LMNA* (1q22) and chromosome 13 in *RB1* (13q14.2) resulted in dicentric and acentric chromosomes, respectively, containing the distal parts of the q arms welded together. This generated truncated proteins with 3′-3′ and 5′-5′ junctions, resulting in loss of function.

Altogether, the genomic profiling data, including mutations, variants of unknown significance (Supplementary Table 2), copy number variants, and gene fusion, clarified that the co-occurring adenocarcinomas were derived from the intraductal precursors and shared with them the majority of somatic alterations. Three cases harbored additional alterations restricted to the invasive components, such as mutations affecting the chromatin remodeling genes *ARID2*, *ASXL1*, and *PBRM1*, observed in three cases, and the *ERBB2*-*P3H4* fusion in the case with *ASXL1* mutation.

**Fig. 2:**
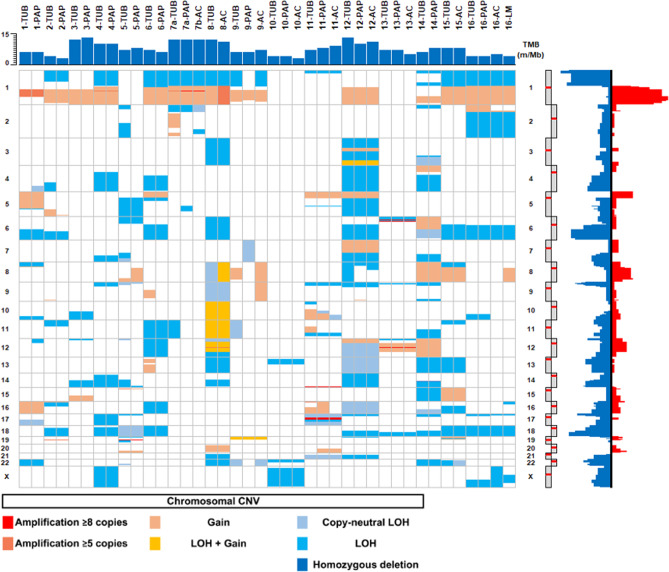
Summarizing figure of the chromosomal alterations identified in all cases. In this figure, chromosomes are presented in increasing order. Note: for chromosomal alterations, the adenocarcinoma of case #14 was not analyzed due to the low cellularity of the sample.

Chromosomal alterations were observed in all samples (Fig. 2). Chromosomal gains were detected in 15 cases (93.7%), whereas chromosomal loss was observed in all cases. The most common alterations were 1q gain, detected in 12 cases (75%), and 1p, 6q, or 18q losses found in 8 (50%), 9 (56.2%), and 9 (56.2%) cases, respectively. Loss of heterozygosity (LOH) followed by reduplication, leading to copy-neutral LOH or LOH with additional gain, was observed in 9 (56.2%) cases.

#### Transcriptome analysis

**Fig. 3:**
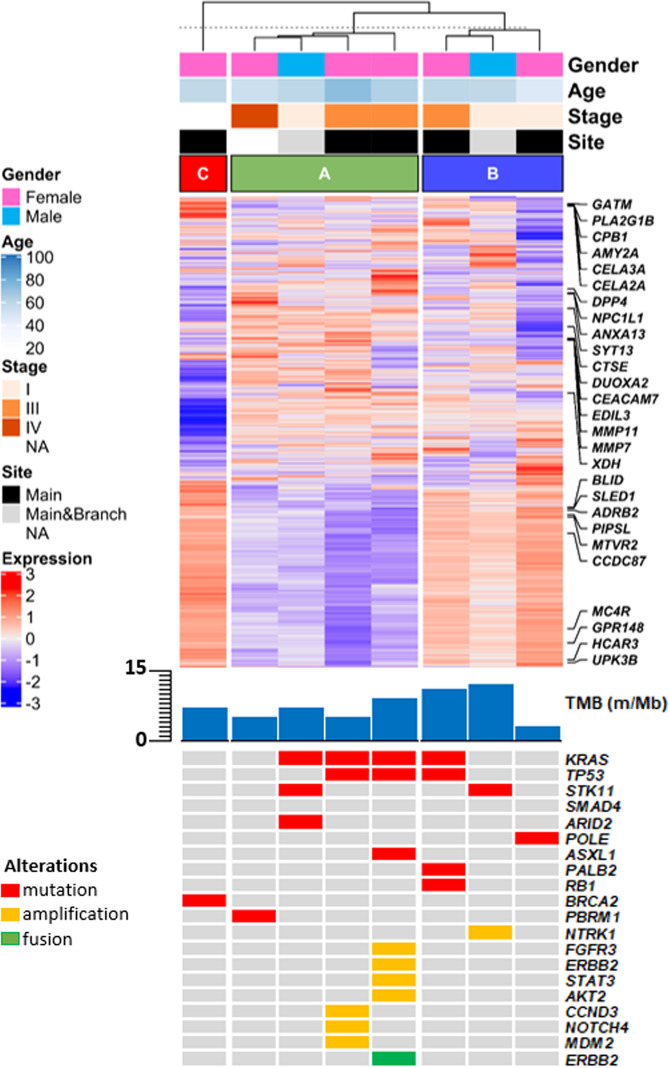
Transcriptome analysis and matched genomic profiles of ITPN-related adenocarcinomas. Upper panel: gene expression heatmap stratified by the three consensus clusters (A, B, and C) derived from the transcriptome analysis of the cohort adenocarcinomas. Annotations for clinicopathologic variables are also provided. Lower panel: genomic alterations for essential tumor-related genes found in all three clusters.

Overall, eight ITPN-concomitant adenocarcinomas were investigated using transcriptome analyses (Fig. 3). A hybrid hierarchical k-means approach (*k* = 3) was used to perform principal component analysis and design a dendrogram showing the relationships between samples. The resulting consensus matrix obtained from the unsupervised consensus clustering confirmed the associations obtained by the principal component analysis and the dendrogram.

The three identified clusters, A, B, and C (Fig. 2), included four and three samples, and one sample, respectively. Pairwise differential expression analysis was performed for all identified clusters. Cluster A showed 21 differentially expressed (DE) genes (Supplementary Table 3), in which no genes with cluster-based statistical significance were identified in clusters B and C. Comparison with the mutational analysis results showed that cluster A was enriched with 3 cases (75%) containing *KRAS* mutations and *ARID2*/*PBMR1* chromatin remodeling. Cluster B included one case (33%) with a *BRAF* mutation. The single case in cluster C showed a *BRCA* alteration (germline *BRCA2* mutation coupled with LOH).

**Fig. 4:**
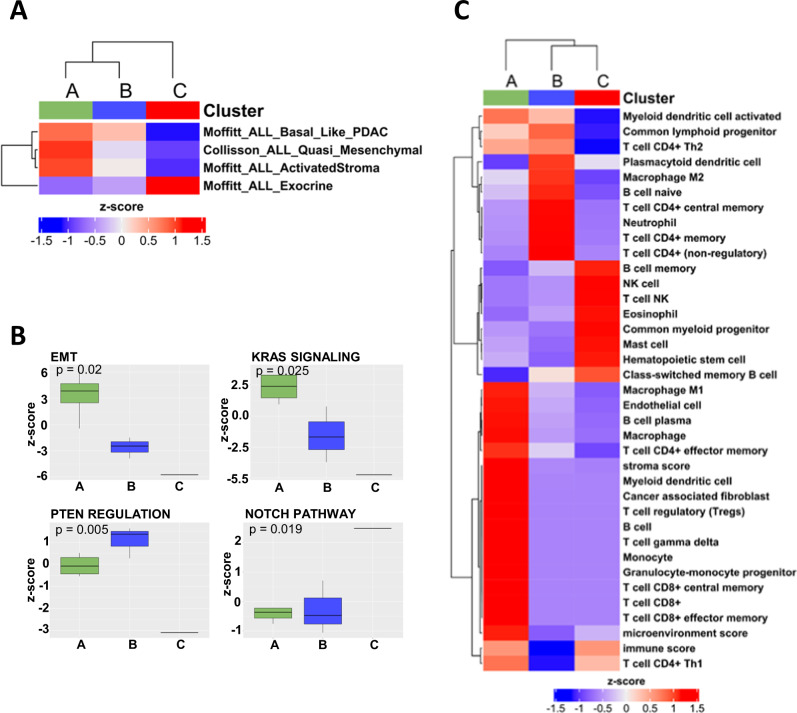
Cluster-based representation of transcriptome analysis. **A** Heatmap showing similar statistically significant transcriptomic profiles among the clusters identified in the current study with the existing molecular subgroups of pancreatic ductal adenocarcinoma (Moffit’s, Collisson’s, and Bailey’s subgroups); **B** Activation of different biological mechanisms in the three clusters. Statistically significant values are shown. **C** Heatmap of the immune subpopulations inferred by gene expression of immune-related metagenes significantly enriched in any of the three clusters.

The comparison between the expression profiles of each cluster and current PDAC classifiers highlighted that cluster A showed squamous-like signatures, similar to Moffitt’s basal-like and active stroma profiles and Collisson’s quasi-mesenchymal profile. In contrast, cluster C showed features of the classical pancreatic subtype similar to the exocrine profile. No statistically significant associations were identified for cluster B; nonetheless, we noted a trend for similarity with Moffitt’s basal-like subgroup (squamous-like profile) (Fig. 4A). Furthermore, using the GSEA-based approach, we identified differential biological processes among the three clusters. Based on the z-score, cluster A showed enrichment for induction of the epithelial-to-mesenchymal (EMT) pathway and KRAS signaling. In contrast, cluster B presented enrichment for the phosphatase and tensin homolog (PTEN) regulation pathways, whereas *NOTCH* signaling was enriched in cluster C (Fig. 4B).

Using deconvolution analysis of the different clusters, statistically significant differences in immune cell populations were identified. Cluster A showed enrichment in CD8+ T-cells, M1-class macrophages, and cancer-associated fibroblasts (CAFs). Cluster B was enriched in CD4+ T-cells and M2-class macrophages, while cluster C was enriched in inflammatory cells implicated in the innate immune response (Fig. 4C).

Chromogenic multiplex IHC for CD4, CD8, and CD163 confirmed these findings, showing predominant CD8+ T-lymphocyte infiltration in cluster A (mean scores: CD8 = 3.8; CD4 = 1.6; CD163 = 1.2), predominance of class 2 macrophages and CD4+ T-lymphocytes in cluster B (mean scores: CD8 = 1.2; CD4 = 3.4; CD163 = 4.2), and low presence of cells positive for these markers in cluster C (mean scores: CD8 = 1.2; CD4 = 1.2; macrophages = 0.8).

#### Integrative multi-regional genomic and transcriptome analysis of ITPN, adenocarcinoma, and liver metastasis

Integrative genomic and transcriptome analyses were performed in the case of metastatic ITPN; in particular, on the tubular and papillary components of ITPN, concomitant pancreatic adenocarcinoma, and liver metastasis. The genomic analysis showed the presence of a truncating mutation in *PBRM1* in the pancreatic adenocarcinoma. In the transcriptome analysis, statistical analysis showed no DE genes between the tubular and papillary components. By comparison, up-regulation of 15 genes and down-regulation of 8 genes were detected in the adenocarcinoma (Supplementary Table 3). The case with the liver metastasis had an even more variable profile: 135 DE genes (113 up-regulated and 22 down-regulated) between the metastatic and intraductal areas and 156 DE genes (103 up-regulated and 53 down-regulated) between the primary and metastatic adenocarcinoma were detected (Supplementary Table 3). On the basis of the highest and statistically significant values of correlation to the current PDAC signatures (Supplementary Fig. 3), the tubular and papillary components were very similar and showed a classical pancreatic profile (Collisson’s classical)^31^. In contrast, the adenocarcinoma showed features of the squamous profile, with positive enrichment for Collisson’s quasi-mesenchymal subtype^31^. By comparison, the transcriptomic profile of liver metastasis showed classical pancreatic features, with positive correlation with Bailey’s immunogenic profile^30^.

### Survival analysis

**Fig. 5:**
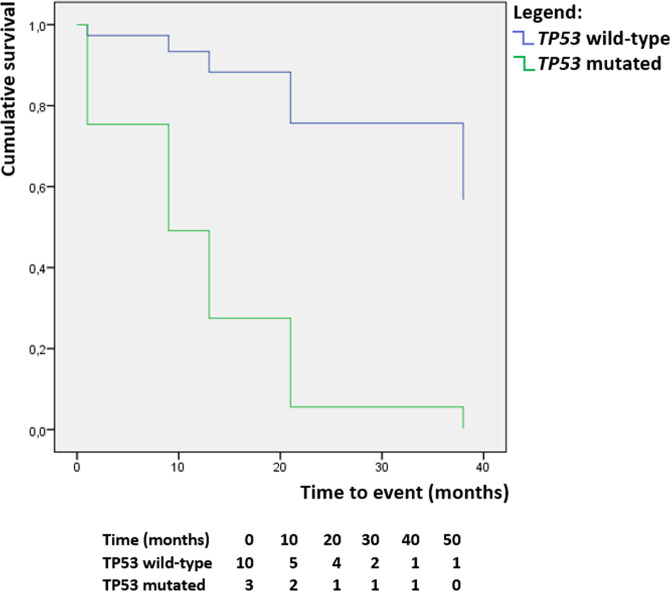
TP53-based survival analysis. Kaplan–Meier curves show patients’ survival in relation to the presence of *TP53* mutation (blue: *TP53* wild type; green: mutated *TP53*).

In the survival analysis, the only parameter that showed statistically significant association with prognostic outcomes was the *TP53* mutation, associated with an increased risk of death or recurrence (hazard ratio = 10.359, 95% confidence interval 1.911–117.776, *p* = 0.039; Fig. 5). The statistical significance of the *TP53* mutation was maintained in the cases with concomitant adenocarcinoma (hazard ratio = 9.569, 95% confidence interval 1.861–106.371, *p* = 0.046).

## Discussion

In this study, we performed a comprehensive characterization of pancreatic ITPN and concomitant invasive adenocarcinoma in 16 cases. Below, we summarize our major findings. 1) Clinicopathologic features: concomitant adenocarcinoma was present in 75% of cases, represented by glandular/tubular adenocarcinomas; 2) ITPN as a precursor of pancreatic cancer: at the molecular level, the co-occurring adenocarcinoma was always associated with pancreatic intraductal components, establishing ITPN as a definitive precursor of pancreatic cancer; 3) tumor progression: mutations of chromatin remodeling genes represented a late event during ITPN oncogenesis. Indeed, mutations affecting such genes have been detected only in the invasive component of three different cases; 4) clinical genetics: ITPN can arise in the context of genetic syndromes, such as HBOC and Peutz–Jeghers, with direct implications for screening, therapy and genetic counseling; 5) mutational profile: mutations in the classical PDAC drivers are less frequent in pancreatic ITPN; 6) copy number variation: recurrent amplifications were observed for the Cyclin (3/16 cases, 18.75%) and *NOTCH* family genes (2/16 cases, 12.5%), whereas *ERBB2*, a potential target for molecular-based therapies, was amplified in one case; 7) chromosomal alterations: the most commonly observed were 1q gain (75% of cases) and 1p, 6q or 18q loss (approximately 50% of cases); 8) structural variations: common fusions involved the recently identified *RET* and *FGFR2*; 9) transcriptome analysis of ITPN-associated adenocarcinoma: three different clusters were identified, with the majority of cases displaying squamous-like features, differential activation of EMT, KRAS-signaling, and PTEN pathways, and variable immune microenvironment composition; and 10) survival analysis: the *TP53* mutational status emerged as a hallmark of adverse prognosis.

At the molecular level, a critical finding emerged from the comparative analysis between intraductal components and concomitant adenocarcinoma: invasive cancers were present in 75% of cases and were always molecularly associated with intraductal components. Indeed, in our case series, all ITPN and co-occurring adenocarcinomas shared most of the genomic alterations. These data provide definitive evidence of ITPN as origin of invasive pancreatic adenocarcinoma. By contrast, a previous study found that co-occurring IPMN and adenocarcinomas were independent (i.e., not molecularly associated) in approximately 20% of cases^34,35^. Interestingly, we found that acquisition of the invasive phenotype in ITPN was always accompanied by alterations in the infiltrative lesion.

Mutations in the chromatin remodeling *ARID2*, *ASXL1* or *PBRM1* were observed only in the invasive component of three different cases. Alterations in the same class of genes have also been reported in the biliary counterpart of these neoplasms^36^; overall, present and previous findings suggest a potential role of this gene class in tumor progression and invasion. Alterations in chromatin remodeling genes have also been reported in the most comprehensive report published to-date on the molecular landscape of pancreatic ITPN^13^. Chromosome 1p loss and 1q gain in the majority of cases are additional common findings. However, some differences between the two studies are evident. First, Basturk et al.^13^ found chromatin remodeling genes with mutations or amplifications in a substantial subset of their cases (30–40% of cases); alterations were relatively rare in our study (approximately 20%). Second, in the earlier study, alterations were commonly detected in *MLL*; no such alterations were observed in our material. Nonetheless, the picture that emerges from these studies confirms that chromosomal alterations and mutations in chromatin remodeling genes are important components in the ITPN molecular landscape, with a potential role in acquiring invasiveness.

This study is the first to report that pancreatic ITPN can arise as part of the spectrum of genetic syndromes, a finding confirmed by molecular analysis. In our cases, neoplasms arose in the context of HBOC syndrome due to *BRCA2* alteration and in the context of Peutz–Jeghers syndrome. Both neoplasms had infiltrative components. These findings have immediate implications for tumor screening and genetic counseling for patients with pancreatic ITPN and may influence clinical management (e.g., platinum-based chemotherapy and PARP-inhibitors for *BRCA*-tumors)^37^. These results emphasize the importance of a thorough anamnesis, including family history of cancer, of all patients presenting with pancreatic ITPN.

The present study confirmed the genomic distinctiveness of ITPN by showing that typical PDAC drivers, including *KRAS*, *TP53*, *SMAD4*, and *CDKN2A*, are less frequently altered in this lesion in comparison with conventional PDAC. Alterations in PDAC drivers, at similar or lower frequency, have already been reported in previous studies of pancreatic ITPNs^38^. The relative paucity of PDAC alterations in this case series highlights the molecular differences with conventional PDAC, but it should be acknowledged that *KRAS* alterations are still present in a not-negligible subset of cases (4/16 cases in this series, 25%). This indicates that pancreatic ITPN cannot be considered as a *KRAS*-independent entity, also taking into account that MAPK-pathway can be activated in this tumor type also through *BRAF* alterations (case #7).

Although the genomic landscape of pancreatic ITPN appears largely heterogeneous, notable common events are represented by gene amplification and fusion. Recurrent amplifications were found in genes of the Cyclin and NOTCH families. Amplification in *ERBB2* in these neoplasms represents a novel finding and a potential target for precision oncology. It should be noted that *ERBB2* is considered one of the most important targets for tailored treatments in breast and gastric cancer^39^, and our findings suggest new promising perspectives in treatment strategies for pancreatic cancer. Gene fusions commonly involved *RET* and *FGFR2*. Fusions involving *FGFR2* have already been reported in two pancreatic ITPNs^13^ but with different partners from those reported here. Conversely, fusions involving *RET* represent a novel finding in pancreatic ITPN, detected here in two cases. Furthermore, we reported a novel *ERBB2*-*P3H4* fusion and a newly established translocation involving *LMNA* and *RB1*, resulting in gene loss. All these detected rearrangements should be considered in molecular-based therapies, already approved for other cancer types^40–42^. The new molecular targets merit particular consideration as potential therapy targets in patients with ITPN-associated pancreatic cancer, especially in the metastatic setting.

Unsupervised clustering of DE genes in ITPN-associated adenocarcinomas identified three different clusters; however, the analysis at this stage should be considered exploratory due to the small sample size. Cluster A showed activation of KRAS signaling and EMT, and displayed squamous features, and enrichment in CD8+ T-cells, M1-class macrophages, and CAFs. Cluster B showed positive correlation with PTEN regulation, similar features to the PDAC squamous-like subgroup, and was enriched with CD4+ T-cells and M2-class macrophages. Cluster C showed activation of NOTCH signaling and a transcriptomic profile toward classical-pancreatic features. Although most cases displayed squamous features, the tumor microenvironment and biological processes activated in the tumors showed substantial differences. These aspects highlight the heterogeneity of tumor microenvironment in pancreatic ITPN and should be considered in future studies to indicate personalized therapeutic approaches^43^. Along these lines, a recent study demonstrated that concurrent loss of *Arid1a* and *Pten* in adult pancreatic ductal cells induced ITPN and ITPN-derived PDAC in mice^44^. In our cohort, the majority of cases studied by transcriptome analysis did show enrichment in the activation of EMT and PTEN-regulation pathway. Moreover, an *ARID*-gene mutation was detected in the invasive component in one case. Overall, our study extend results from animal studies to human disease and confirms the role of *PTEN* and *ARID* in pancreatic ITPN and associated cancers.

Interestingly, the analysis of a primary ITPN coupled with invasive and metastatic sites highlighted that the pancreatic transcriptional program can be plastic across different tumor stages. Despite genomic relatedness, the intraductal components featured the classical pancreatic subtype, whereas squamous-like characteristics were presented in the invasive adenocarcinoma and classical-pancreatic features in distant metastasis. This finding can be best appreciated in view of recent pioneering studies that found evidence of subtype switching during tumor progression^45–47^. Although the mechanism in PDAC is still not fully understood, our initial analysis of a pancreatic ITPN case and the associated primary and metastatic adenocarcinoma suggests that subtype switching may be necessary for intraductal lesions to acquire infiltrating and further metastatic capability^48^.

Finally, a finding that merits attention is the role of *TP53* mutational status in adverse prognosis; importantly, the *TP53* mutational status was maintained in the multivariable analysis that comprised cases with invasive adenocarcinoma. Association of *TP53* mutations with an adverse prognosis is commonly encountered in different cancer types, including colorectal and ampullary adenocarcinomas in the gastrointestinal tract^49,50^. This finding may help stratify patients with ITPN at diagnosis. The *TP53* mutational status could, thus, be adopted as a potential prognostic biomarker to identify high-risk lesions requiring aggressive therapeutic and surgical strategies. As demonstrated here, IHC is a valuable supportive tool for detecting *TP53*-mutated cases; potential applications of IHC in detecting this biomarker during routine diagnostic activity could be adopted.

It is important to acknowledge that this study has some limitations. First, the genomic analysis did not investigate the whole genome of the lesions; thus, potentially significant molecular events could have been missed. Nonetheless, the CORE panel we adopted was based on previously reported whole-genome sequencing focused on clinically relevant alterations. Furthermore, although the results of the transcriptomic analysis represent a novelty in the ITPN-context, they are based on eight cases and should be considered as exploratory rather than conclusive. We must also acknowledge that, despite the relatively small sample size, the multicenter design of the current study is a concrete answer to the difficulties of collecting large case series of rare neoplasms.

In conclusion, in this study we provided an integrative clinicopathologic and molecular characterization of a series of pancreatic ITPNs and associated adenocarcinomas. Our findings highlight that these lesions represent a distinct entity among pancreatic neoplasms. In the context of pancreatic intraductal/cystic lesions, correct identification of ITPNs is crucial given their distinctive clinicopathologic features, genomic and transcriptomic profiles, and potential for target-enrichment strategies for precision oncology.

## Supplementary information

Supplementary Material

## Data Availability

All data/information are available in the manuscript and in the Supplementary Material.
