# Supplementary material for: Integrative characterization of intraductal tubulopapillary neoplasm (ITPN) of the pancreas and associated invasive adenocarcinoma
Source: Mod Pathol. 2022 Sep 2;35(12):1929–43. doi: 10.1038/s41379-022-01143-2 (PMC9708572; doi:10.1038/s41379-022-01143-2)

SUPPLEMENTARY MATERIAL

Supplementary Table 1. Targeted genes in the CORE sequencing assay.

Supplementary Table 2. Variants of unknown significance (VUS) of pancreatic ITPN and concomitant invasive carcinoma.

Supplementary Table 3. Differentially expressed genes based on the transcriptomic analysis.

Supplementary Figure 1. Representative immunohistochemical images of p53 staining in cases harboring *TP53* mutations (10x magnification; A: case #5; B: case #11; C: case #12; D: case #13). All cases showed diffuse nuclear positivity (>90% of neoplastic cells).

Supplementary Figure 2. Representative images of the adenocarcinoma harboring *ERBB2* fusion and amplification. (A) Hematoxylin-eosinstained histological slides (20x magnification). (B) Immunohistochemistry for Her2 showing a heterogeneous staining pattern, from a weak to a focally strong positivity.

Supplementary Figure 3. Summarizing figure of the pancreatic ITPN case with associated adenocarcinoma and liver metastasis. Upper panel: Heatmap showing the statistically significant similarities in the transcriptomic profiles of the different tumor components (TUB: intraductal tubular component; PAP: intraductal papillary; AC: adenocarcinoma; LM: liver metastasis) with the existing molecular subgroups of pancreatic ductal adenocarcinoma (Moffit’s, Collisson’s and Bailey’s subgroups). Lower panel: Hematoxylin-eosin images illustrating the (A) intraductal component (10x magnification); (B) papillary component (10x magnification); (C) invasive adenocarcinoma (4x magnification); and (D) liver metastasis (10x magnification). Asterisk indicates the normal liver parenchyma, and hash symbol indicates the metastatic lesion.

Supplementary Table 1. Targeted genes in the CORE sequencing assay

AKT1	*B2M*	*CDKN1A*	*ERG*	*HIST1H3B*	*KMT2A*	*MYB*	*PDGFRB*	*RHOA*	*STAT5B*
AKT2	*BAP1*	*CDKN1B*	*ESR1*	*HIST1H3C*	*KRAS*	*MYC*	*PHF6*	*RNF43*	*STK11*
AKT3	*BCL2*	*CDKN2A*	***ETV6****	*HIST2H3C*	*MAP2K1*	*MYCN*	*PIK3CA*	***ROS1***	*SYK*
ALK	*BLM*	*CDKN2B*	*EZH2*	*HLA-A*	*MAP2K2*	*NBN*	*PIK3CB*	*RPL5*	***TERT***
AMER1	***BRAF***	*CHEK2*	*FAS*	*HLA-B*	*MAP2K4*	*NF1*	*PIK3R1*	*RUNX1*	*TGFBR2*
APC	*BRCA1*	*CIITA*	*FBXW7*	*HLA-C*	*MAP3K1*	*NF2*	*PMS2*	*SETBP1*	*TP53*
APLNR	*BRCA2*	*CREBBP*	*FGF19*	*HNF1A*	*MAPK1*	*NFE2L2*	*POLE*	*SETD2*	*TSC1*
AR	*CBL*	*CTCF*	*FGFR1*	*HRAS*	*MAX*	*NOTCH1*	*POLQ*	*SF3B1*	*TSC2*
ARAF	*CCND1*	*CTNNB1*	***FGFR2***	*IDH1*	*MCL1*	*NOTCH2*	*PPP2R1A*	*SMAD4*	*U2AF1*
ARID1A	*CCND2*	*DAXX*	***FGFR3***	*IDH2*	*MDM2*	*NOTCH3*	*PTCH1*	*SMARCA4*	*VHL*
ARID1B	*CCND3*	*DICER1*	*FGFR4*	*IGF1R*	*MED12*	*NOTCH4*	*PTEN*	*SMARCB1*	*WT1*
ARID2	*CCNE1*	*DNMT3A*	*GATA3*	*JAK1*	*MEN1*	*NPM1*	*PTPN11*	*SMO*	*YAP1*
ASXL1	*CD274*	***EGFR***	*GNA11*	*JAK2*	*MET*	*NRAS*	*RAC1*	*SOCS1*	
ATM	*CD58*	*EP300*	*GNAQ*	*JAK3*	*MLH1*	***NTRK1***	*RAD21*	*SPOP*	
ATR	*CDK12*	*EPHA3*	*GNAS*	*JUN*	*MSH2*	*PALB2*	*RAD50*	*SRC*	
ATRX	*CDK2*	*ERBB2*	*H3F3A*	*KDR*	*MSH6*	*PBRM1*	***RAF1***	*STAG1*	
AURKA	*CDK4*	*ERBB3*	*H3F3B*	*KIT*	*MTOR*	*PDCD1LG2*	*RB1*	*STAG2*	
AXL	*CDK6*	*ERBB4*	*HGF*	*KLF4*	*MUTYH*	*PDGFRA*	***RET***	*STAT3*	

Coding exons | Coding and copy number | *Copy number only* | Full gene footprint for structural variants | **Fusions detected**

* ETV6 reports NTRK3 fusions

Supplementary Table 2: Variants of unknown significance (VUS) of pancreatic ITPN and concomitant invasive carcinoma

ID case	Histology	Variants of unknown significance (VUS)				
		**Gene**	**Variation**	**Mutation type**	**Freq (%)**	**Class**
1	Tubular	*CREBBP*	p.L795F	Substitution - missense	36	3
		PBRM1	L693_P694InsM	Inframe insertion	16	3
		RNF43	p.T64P	Substitution - missense	75	3
	Papillary	*CREBBP*	p.L795F	Substitution - missense	35	3
		PBRM1	L693_P694InsM	Inframe insertion	51	3
		RNF43	p.T64P	Substitution - missense	71	3
2	Tubular	*NBN*	c.171+4T>C	Substitution - splice site	46	3
		WT1	p.Q410H	Substitution - missense	18	3
	Papillary	*NBN*	c.171+4T>C	Substitution - splice site	58	3
		WT1	p.Q410H	Substitution - missense	16	3
3	Tubular	*ESR1*	p.V392I	Substitution - missense	44	3
		FGFR4	p.N624T	Substitution - missense	47	3
		GNAS	p.V636I	Substitution - missense	53	3
		NOTCH3	p.V2202I	Substitution - missense	32	3
		NOTCH4	p.G1790A	Substitution - missense	44	3
		PALB2	p.I922V	Substitution - missense	40	3
		RPL5	p.A148G	Substitution - missense	32	3
	Papillary	*ESR1*	p.V392I	Substitution - missense	40	3
		FGFR4	p.N624T	Substitution - missense	52	3
		GNAS	p.V636I	Substitution - missense	46	3
		NOTCH3	p.V2202I	Substitution - missense	29	3
		NOTCH4	p.G1790A	Substitution - missense	31	3
		PALB2	p.I922V	Substitution - missense	35	3
		RPL5	p.A148G	Substitution - missense	26	3
4	Tubular	*GNAS*	p.S313R	Substitution - missense	19	3
	Papillary	*GNAS*	p.S313R	Substitution - missense	17	3
5	Tubular	*MTOR*	p.R1611Q	Substitution - missense	36	3
		POLE	p.V1426I	Substitution - missense	29	3
	Papillary	*MTOR*	p.R1611Q	Substitution - missense	13	3
		POLE	p.V1426I	Substitution - missense	15	3
6	Tubular	*ATM*	p.R1489C	Substitution - missense	80	3
		NOTCH1	p.A585T	Substitution - missense	50	3
		RAD21	p.E495D	Substitution - missense	44	3
		MEN1	p.D597A	Substitution - missense	91	3
		MED12	p.P1860H	Substitution - missense	35	3
	Papillary	*ATM*	p.R1489C	Substitution - missense	66	3
		NOTCH1	p.A585T	Substitution - missense	50	3
		RAD21	p.E495D	Substitution - missense	45	3
		MEN1	p.D597A	Substitution - missense	84	3
7a*	Tubular	*ATM*	p.A2040G	Substitution - missense	26	3
		GNAS	p.R246H	Substitution - missense	34	3

		JAK1	p.F837L	Substitution - missense	37	3
		MUTYH	p.C262Y	Substitution - missense	78	3
		PTCH1	p.R1113C	Substitution - missense	48	3
	Papillary	*ARID1A*	p.F1720L	Substitution - missense	5	3
		ATM	p.A2040G	Substitution - missense	53	3
		GNAS	p.R246H	Substitution - missense	35	3
		MUTYH	p.C262Y	Substitution - missense	72	3
		PTCH1	p.R1113C	Substitution - missense	47	3
7b*	AC	*ATM*	p.A2040G	Substitution - missense	47	3
		GNAS	p.R246H	Substitution - missense	26	3
		MUTYH	p.C262Y	Substitution - missense	62	3
		PTCH1	p.R1113C	Substitution - missense	47	3
8	Tubular	*JAK2*	p.R923H	Substitution - missense	29	3
		RAD50	p.R83G	Substitution - missense	50	3
		SMARCA4	p.V343A	Substitution - missense	50	3
	AC	*JAK2*	p.R923H	Substitution - missense	27	3
		RAD50	p.R83G	Substitution - missense	49	3
		SMARCA4	p.V343A	Substitution - missense	48	3
9	Tubular	*ERBB2*	c.226-5C>T	Substitution - intronic	42	3
		NOTCH1	p.A683T	Substitution - missense	49	3
		MET	p.I662V	Substitution - missense	59	3
		NFE2L2	p.A51Rfs*7	Substitution - frameshift	9	3
	Papillary	*ERBB2*	c.226-5C>T	Substitution - splice site	43	3
		NOTCH1	p.A683T	Substitution - missense	41	3
		MET	p.I662V	Substitution - missense	58	3
	AC	*ERBB2*	c.226-5C>T	Substitution - intronic	49	3
		NOTCH1	p.A683T	Substitution - missense	35	3
		MET	p.I662V	Substitution - missense	73	3
10	Tubular	*MET*	p.A8T	Substitution - missense	37	3
		SETD2	p.T1220N	Substitution - missense	36	3
		AR	c.1886-4A>G	Substitution - splice site	65	3
	Papillary	*MET*	p.A8T	Substitution - missense	43	3
		SETD2	p.T1220N	Substitution - missense	41	3
		AR	c.1886-4A>G	Substitution - splice site	72	3
	AC	*MET*	p.A8T	Substitution - missense	35	3
		SETD2	p.T1220N	Substitution - missense	43	3
		AR	c.1886-4A>G	Substitution - splice site	58	3
11	Tubular	*MSH6*	p.A1302T	Substitution - missense	54	3
	Papillary	*CCNE1*	p.Q197H	Substitution - missense	27	3
		MSH6	p.A1302T	Substitution - missense	49	3
	AC	*MSH6*	p.A1302T	Substitution - missense	30	3
12	Tubular	*DAXX*	c.815-5C>T	Substitution - splice site	49	3
		KDR	p.Q441E	Substitution - missense	26	3
		PDGFRA	p.A7V	Substitution - missense	23	3
		SF3B1	p.R238C	Substitution - missense	59	3

	Papillary	*DAXX*	c.815-5C>T	Substitution - splice site	49	3
		PDGFRA	p.A7V	Substitution - missense	16	3
		SF3B1	p.R238C	Substitution - missense	32	3
	AC	*DAXX*	c.815-5C>T	Substitution - splice site	49	3
		KDR	p.Q441E	Substitution - missense	5	3
		PDGFRA	p.A7V	Substitution - missense	30	3
		SF3B1	p.R238C	Substitution - missense	57	3
13	Tubular	*AR*	p.G416S	Substitution - missense	53	3
		CDKN2A	p.D153N	Substitution - missense	29	3
	Papillary	*AR*	p.G416S	Substitution - missense	48	3
		CDKN2A	p.D153N	Substitution - missense	45	3
	AC	*AR*	p.G416S	Substitution - missense	50	3
		CDKN2A	p.D153N	Substitution - missense	9	3
14	Tubular	*POLE*	p.E1963Q	Substitution - missense	41	3
	Papillary	*POLE*	p.E1963Q	Substitution - missense	37	3
	AC	*POLE*	p.E1963Q	Substitution - missense	7	3
15	Tubular	*EZH2*	p.R213H	Substitution - missense	36	3
		KRAS	p.V112I	Substitution - missense	52	3
		POLQ	p.V1701I	Substitution - missense	46	3
	AC	*EZH2*	p.R213H	Substitution - missense	36	3
		KRAS	p.V112I	Substitution - missense	43	3
		POLQ	p.V1701I	Substitution - missense	41	3
16	Tubular	*ALK*	p.F270L	Substitution - missense	47	3
		ATR	p.H453Q	Substitution - missense	42	3
		DAXX	p.R19H	Substitution - missense	30	3
		ATM	p.L2492R	Substitution - missense	53	3
		NOTCH3	p.T411K	Substitution - missense	50	3
	Papillary	*ALK*	p.F270L	Substitution - missense	48	3
		ATR	p.H453Q	Substitution - missense	46	3
		DAXX	p.R19H	Substitution - missense	23	3
		ATM	p.L2492R	Substitution - missense	48	3
		NOTCH3	p.T411K	Substitution - missense	50	3
	AC	*NTRK1*	p.V475M	Substitution - missense	11	3
		ALK	p.F270L	Substitution - missense	45	3
		ATR	p.H453Q	Substitution - missense	45	3
		ATM	p.L2492R	Substitution - missense	49	3
		ERBB3	p.D628Y	Substitution - missense	13	3
		NOTCH3	p.T411K	Substitution - missense	49	3
	Liver met.	*ALK*	p.F270L	Substitution - missense	51	3
		ATR	p.H453Q	Substitution - missense	53	3
		ATM	p.L2492R	Substitution - missense	63	3
		NOTCH3	p.T411K	Substitution - missense	60	3

Legend and abbreviations: AC: adenocarcinoma; met: metastasis. Class: clinical Impact class according to AMGP/AMP guidelines (3: variant of unknown significance; Richards *et al.* Genet Med 2015).
Notes: *7a pancreatic resection for ITPN; 7b: local relapse as adenocarcinoma.

Supplementary Table 3. Differentially expressed genes based on the transcriptomic analysis.

1. MAIN CLUSTERS

Differentially expressed (DE) genes characterizing cluster A (alphabetical order) and reaching the statistical significance. No statistically significant DE genes characterized clusters B and C.

	Cluster A vs. Cluster B		Cluster A vs. Cluster C	
Genes	Log_2 Fold Change	Adjusted p-value	Log_2 Fold Change	Adjusted p-value
ANXA13	4,105672515	0,020614	5,532819	0,029647
C12orf36	3,150162369	0,048935	4,676995	0,038844
CEACAM7	3,852888982	0,039507	5,355246	0,039809
CTSE	5,136971578	0,01653	5,056455	0,028476
CXCL14	3,371983358	0,016614	5,476291	0,028272
CYP2C18	3,38683818	0,04137	6,35483	0,028272
CYP2C19	2,716559126	0,048335	5,410179	0,028272
DPP4	2,565562139	0,049221	5,492023	0,028272
DUOXA2	3,413054335	0,02298	4,415131	0,03202
EDIL3	2,736805436	0,024268	3,39782	0,037501
F5	3,987278528	0,026648	4,800849	0,044896
HTRA3	2,940371002	0,032754	4,693601	0,028518
KRT23	4,357223902	0,019445	5,913799	0,028549
MB21D2	2,680463846	0,024279	3,150186	0,044142
MMP1	4,106308926	0,016717	5,58348	0,028272
MMP11	2,997766866	0,01653	3,349507	0,032352
MMP7	3,02699049	0,019788	3,231568	0,048495
MS4A8B	2,992302058	0,036891	4,299446	0,034597
NPC1L1	3,873110425	0,02004	4,686681	0,033787
SYT13	3,075534174	0,036906	4,210573	0,039686
XDH	3,16347316	0,032215	4,55949	0,031892

2. CASE WITH INTRADUCTAL COMPONENTS, INVASIVE ADENOCARCINOMA AND LIVER METASTASIS

A. DE genes between the intraductal components and the adenocarcinoma (in decreasing order of log Fold Change coefficient of association) and reaching the statistical significance.

Up-regulated genes in the adenocarcinoma	Log_2 Fold Change	Adjusted p-value	Down-regulated genes in the adenocarcinoma	Log_2 Fold Change	Adjusted p-value
CFTR	4,621954	0,048338	*HLA-C*	-1,17734	0,048338
INS	2,672997	0,048338	*HLA-A*	-1,17981	0,048338
SERPINE1	2,499296	0,048338	*LRMP*	-1,27573	0,048338
C7	2,240478	0,048338	*HPD*	-1,52255	0,048338
CTGF	2,133908	0,048338	*APOL3*	-1,56139	0,048338
CCL2	2,005431	0,048338	*CYP2C8*	-1,68489	0,048338
HSD3BP4	1,946557	0,048338	*APOL6*	-1,8179	0,048338
IGFBP7	1,808263	0,048338	*BST2*	-1,83024	0,048338
HBA2	1,789848	0,048338			
PFN1P2	1,693262	0,048338			
UG0898H09	1,691375	0,048338			
OR10R2	1,691375	0,048338			
FBN1	1,611955	0,048338			

GAS5	1,539741	0,048338			
SULF1	1,53657	0,048338			

B. DE genes between the intraductal components and the liver metastasis (in decreasing order of log Fold Change coefficient of association) and reaching the statistical significance.

Up-regulated genes in the metastasis	Log_2 Fold Change	Adjusted p-value	Down-regulated genes in the metastasis	Log_2 Fold Change	Adjusted p-value
HP	7,017802	0,005943	*SNORA23*	-1,64489	0,013022
APOA1	6,0387	0,005943	*ZFP36*	-1,7306	0,015592
ALB	5,51791	0,009977	*ATF3*	-1,73175	0,047397
APOA2	5,318341	0,005943	*SNORD15A*	-1,75088	0,009977
FGB	5,190086	0,016441	*SNORA50*	-1,76231	0,018358
TF	5,102526	0,009927	*DUOX1*	-1,80858	0,008987
FGG	4,759302	0,011371	*SNORA5A*	-1,89194	0,044767
APOH	4,596138	0,005943	*DUSP1*	-1,93142	0,017044
ALDOB	4,586933	0,005943	*SNORD15B*	-1,93231	0,01775
CYP3A4	4,45091	0,006448	*SNORA46*	-1,93317	0,007609
APOC2	4,370468	0,009977	*ST8SIA3*	-1,981	0,048067
PLG	4,336711	0,005943	*FOS*	-2,15996	0,037161
CYP2E1	4,289854	0,008176	*IFI6*	-2,19958	0,025494
ITIH3	4,009893	0,005943	*PDZK1IP1*	-2,24968	0,033056
HRG	4,005914	0,008987	*SLC28A3*	-2,26334	0,027096
APOC3	3,92186	0,00596	*SUCNR1*	-2,49888	0,017882
CPS1	3,67548	0,00596	*S100A1*	-2,89604	0,005943
APOB	3,648244	0,016441	*PI3*	-3,10463	0,017882
ACSM2B	3,621242	0,005943	*GLT25D2*	-3,19635	0,016597
ODAM	3,604911	0,01973	*PIGR*	-3,32447	0,005943
CFHR2	3,511698	0,006448	*TFF1*	-3,53618	0,007447
KNG1	3,427344	0,035757	*TFF2*	-4,67091	0,03076
APOC1	3,406015	0,005943			
CXCL6	3,38009	0,006448			
SERPINC1	3,294735	0,005943			
SERPIND1	3,118743	0,007467			
ADH4	3,118613	0,005943			
HPX	3,114094	0,008751			
C9	3,1099	0,005943			
HSD17B13	3,058271	0,005943			
MAT1A	3,057807	0,007609			
IGFBP1	3,012305	0,005943			
FABP1	2,935021	0,034059			
ADH1B	2,916313	0,015592			
HAO1	2,900423	0,006448			
HPR	2,786892	0,005943			
ANGPTL3	2,785782	0,008176			
AOX1	2,731914	0,0192			
TAT	2,685163	0,006915			
UGT2B4	2,683203	0,008481			
CFHR1	2,60718	0,01514			
ARG1	2,598271	0,005943			
CES1	2,585309	0,008481			

ITIH1	2,50937	0,044767			
F2	2,508368	0,017882			
SLCO1B1	2,472614	0,009977			
CRHBP	2,463856	0,008481			
GSTA1	2,425822	0,01369			
HAMP	2,413042	0,025494			
AFM	2,41261	0,006448			
A1BG	2,405813	0,005943			
F12	2,389296	0,017882			
OLFM4	2,383347	0,021192			
LAPTM4B	2,367651	0,032758			
CYP1A2	2,324755	0,005943			
CYP2A6	2,302911	0,005943			
SULT2A1	2,302911	0,005943			
SEMA3E	2,297183	0,044767			
DMBT1	2,288829	0,035832			
SERPINA11	2,254855	0,005943			
APOF	2,228493	0,005943			
CFHR5	2,22182	0,005943			
UGT2B15	2,211883	0,00612			
AQP9	2,202645	0,017044			
APOE	2,181412	0,008442			
TDO2	2,179307	0,007467			
HSD17B6	2,179278	0,020018			
PZP	2,170526	0,008481			
PON3	2,129872	0,012932			
AKR1C1	2,117191	0,020194			
SLC22A1	2,115137	0,005943			
ACSM2A	2,104572	0,009977			
GABRP	2,101882	0,033056			
UGT2B10	2,080655	0,005943			
UGT2A3	2,070889	0,005943			
FMO3	2,067054	0,009464			
UGT2B7	2,050656	0,012822			
LECT2	1,971777	0,005943			
PON1	1,96918	0,019353			
MT1G	1,917408	0,009977			
F13B	1,905095	0,005943			
CFHR4	1,897272	0,009977			
CCL16	1,883508	0,005943			
MTTP	1,8571	0,005943			
SPP2	1,852643	0,005943			
ANG	1,835842	0,02332			
AADAC	1,834305	0,018358			
C8B	1,830118	0,016441			
APOM	1,82614	0,008481			
ALDH1A2	1,805242	0,03947			
AKR1C2	1,805194	0,02761			
PLA2G2A	1,804854	0,012882			
GGH	1,77894	0,011743			
GLYAT	1,769535	0,00596			

MASP2	1,769535	0,00596			
SLC13A5	1,730697	0,00612			
KIAA0101	1,729029	0,011823			
ACAT1	1,717924	0,044767			
F9	1,695653	0,00612			
AKR1D1	1,685448	0,00612			
RDH16	1,681386	0,014503			
CDO1	1,670928	0,036807			
DPYS	1,659547	0,006225			
ADH1A	1,656124	0,009977			
CYP2B6	1,646728	0,009977			
BAALC	1,646283	0,013011			
C3P1	1,618233	0,03521			
PIPOX	1,616108	0,025494			
MASP1	1,614454	0,028536			
TFAP2A	1,612567	0,007447			
AGMO	1,598069	0,033254			
FCN2	1,597922	0,021597			
FGA	1,57524	0,030464			

C. DE genes between the adenocarcinoma and the liver metastasis (in decreasing order of log Fold Change coefficient of association) and reaching the statistical significance.

Up-regulated genes in the metastasis	Log_2 Fold Change		Down-regulated genes in the metastasis	Log_2 Fold Change	
HP	6,813554	0,024068	*HLA-A*	-0,45948	0,041992
APOA1	5,86448	0,02049	*IGFBP7*	-0,67278	0,036014
APOA2	5,165234	0,034238	*HLA-C*	-0,74716	0,028795
ALDOB	4,812635	0,015876	*PFN1P2*	-0,77312	0,030448
TF	4,679343	0,029185	*UG0898H09*	-0,81351	0,026034
CYP3A4	4,594938	0,037447	*C7*	-0,8236	0,036357
APOH	4,520202	0,025666	*OR10R2*	-0,83688	0,026034
PLG	4,336711	0,015876	*HSD3BP4*	-0,89239	0,025666
HRG	4,335972	0,015961	*COL14A1*	-1,06461	0,045941
KNG1	4,05667	0,033566	*PTGIS*	-1,07581	0,036211
APOC2	4,040388	0,040233	*GOLM1*	-1,1372	0,024068
CYP2E1	3,992412	0,026034	*CASR*	-1,24446	0,043101
ITIH3	3,96069	0,015876	*LCN2*	-1,33966	0,042142
APOC3	3,711337	0,015876	*SIK1*	-1,36441	0,019229
ACSM2B	3,704603	0,028795	*AEBP1*	-1,37833	0,029194
CPS1	3,494854	0,017993	*CPXM2*	-1,48842	0,031693
APOC1	3,439309	0,028795	*F3*	-1,61732	0,027145
FABP1	3,423739	0,0468	*SNORD15A*	-1,70212	0,047862
CFHR2	3,301174	0,015876	*DUSP1*	-1,7034	0,015876
SERPINC1	3,294735	0,015876	*USP17L2*	-1,71201	0,028795
CXCL6	3,182959	0,017371	*DUOX1*	-1,72022	0,034227
IGFBP1	3,132712	0,017371	*SNORA23*	-1,74838	0,043162
ODAM	3,118929	0,033566	*MATN3*	-1,85243	0,045439
ADH4	3,118613	0,015876	*IFI6*	-1,85765	0,015876
C9	3,1099	0,015876	*RGS5*	-1,87514	0,045941

HSD17B13	3,058271	0,015876	*SLC28A3*	-1,91784	0,041992
HAO1	2,983785	0,034707	*GCNT3*	-1,94912	0,031987
ANGPTL3	2,974461	0,017993	*SNORA46*	-1,99044	0,032408
HPX	2,974257	0,045941	*RGCC*	-2,0703	0,017371
SERPIND1	2,90822	0,015876	*CFC1B*	-2,12718	0,045941
UGT2B4	2,874414	0,015901	*FOSB*	-2,15567	0,017993
MAT1A	2,847284	0,015876	*THBS4*	-2,25484	0,048871
F2	2,825845	0,015876	*ST8SIA3*	-2,37747	0,048871
HPR	2,786892	0,015876	*CST1*	-2,37855	0,017114
APCS	2,755943	0,025666	*SERPINE1*	-2,44186	0,015901
HAMP	2,739826	0,0468	*EMP1*	-2,46722	0,04426
HPD	2,707689	0,015876	*SNORA74A*	-2,54596	0,015876
TAT	2,619515	0,037873	*FOS*	-2,5757	0,015876
ARG1	2,598271	0,015876	*SPARCL1*	-2,68494	0,029185
ADH1B	2,584784	0,025666	*CTGF*	-2,70936	0,015876
SLCO1B1	2,580463	0,048449	*SNORD94*	-2,78056	0,037447
CES1	2,440309	0,036357	*GLT25D2*	-2,8032	0,016375
AFM	2,437653	0,035457	*IGJ*	-2,80874	0,026034
A1BG	2,405813	0,015876	*S100A1*	-2,81775	0,025666
AOX1	2,386859	0,039047	*HBA2*	-2,98706	0,015876
CYP1A2	2,324755	0,015876	*DUOX2*	-3,07071	0,037447
CYP2A6	2,302911	0,015876	*PIGR*	-3,17774	0,017993
SULT2A1	2,302911	0,015876	*APOD*	-3,17946	0,032408
CRHBP	2,291863	0,017993	*INS*	-3,27278	0,015901
SAA4	2,290103	0,019229	*IGLL5*	-3,50847	0,048871
SERPINA11	2,254855	0,015876	*TFF1*	-3,66612	0,037873
UGT2B7	2,239335	0,024068	*TFF2*	-3,88718	0,046129
UGT2B15	2,235146	0,029194	*GCG*	-5,90701	0,041992
APOF	2,228493	0,015876			
CFHR5	2,22182	0,015876			
ACSM2A	2,187933	0,045439			
GSTA1	2,16824	0,015876			
SLC22A1	2,130207	0,015876			
UGT2A3	2,110521	0,015876			
ANG	2,095057	0,025607			
TDO2	2,085072	0,029185			
UGT2B10	2,080655	0,015876			
APOE	2,053837	0,026034			
PCK1	2,05048	0,043341			
C8B	2,027775	0,025666			
PZP	2,021515	0,015876			
LECT2	1,971777	0,015876			
AQP9	1,943838	0,026444			
FMO3	1,927009	0,02651			
PON3	1,919349	0,015876			
F13B	1,905095	0,015876			
C3P1	1,892188	0,037729			
CCL16	1,883508	0,015876			
HSD17B6	1,882142	0,015876			
DKK1	1,862571	0,049074			
MTTP	1,8571	0,015876			

SPP2	1,852643	0,015876			
PIPOX	1,852441	0,024309			
CYP2C8	1,770427	0,016375			
GLYAT	1,769535	0,015876			
MASP2	1,769535	0,015876			
MT1G	1,768397	0,015876			
CYP2B6	1,751085	0,026945			
APOM	1,750478	0,034107			
CFHR4	1,748261	0,015876			
SLC13A5	1,730697	0,015876			
GABRP	1,728674	0,024068			
F9	1,695653	0,015876			
AKR1D1	1,685448	0,015876			
TFAP2A	1,662726	0,016375			
DPYS	1,659547	0,015876			
H19	1,554439	0,025666			
ADH1A	1,550198	0,025666			
BAALC	1,497272	0,016375			
FN1	1,343733	0,041992			
C4BPB	1,199459	0,036357			
NT5DC2	1,088281	0,037729			
CXCL12	0,915005	0,041992			
BST2	0,912483	0,025666			
BCHE	0,81596	0,026034			
APOL6	0,783419	0,026801			
FOXQ1	0,76549	0,027145			
PTGR1	0,724359	0,036014			

Supplementary Figure 1.

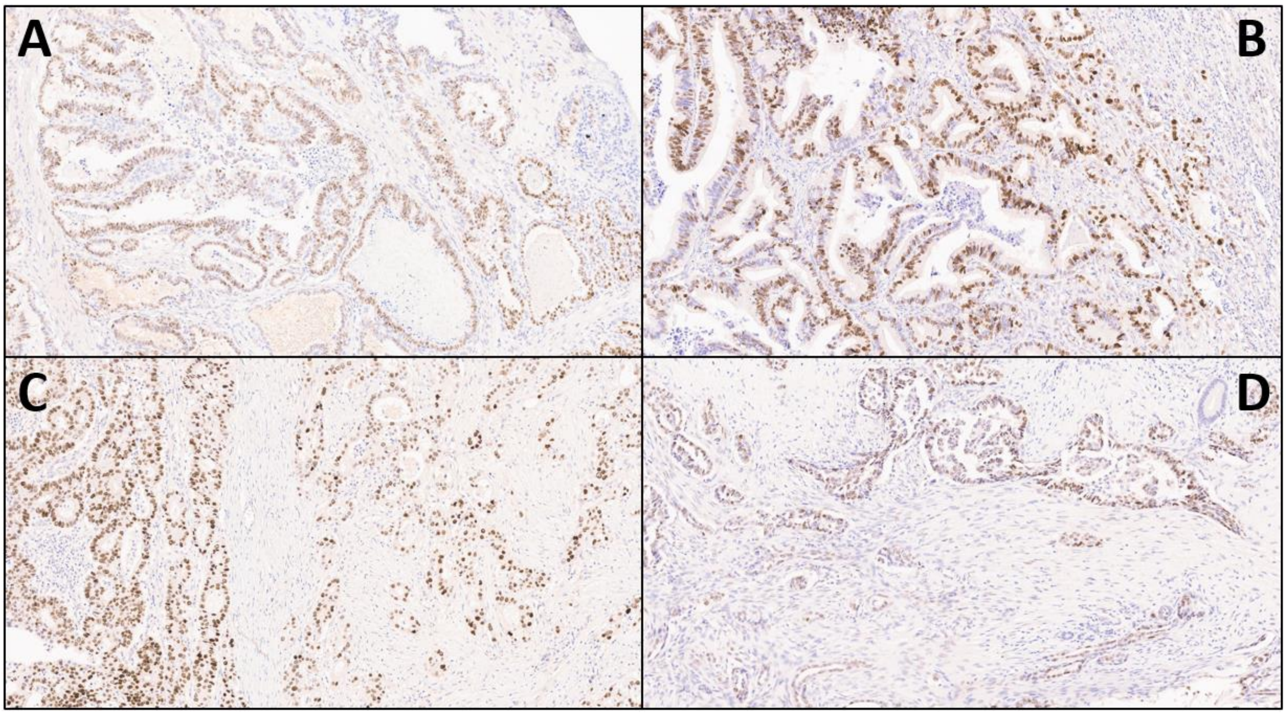

Supplementary Figure 2.

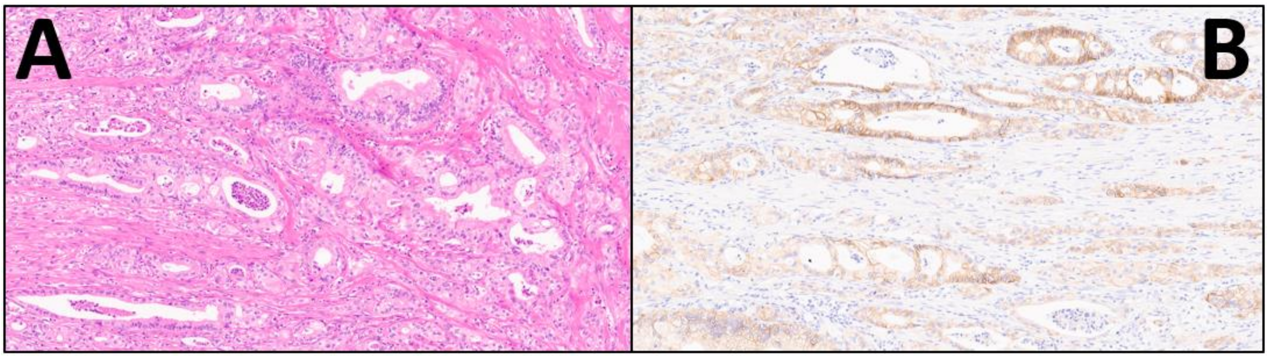

Supplementary Figure 3.

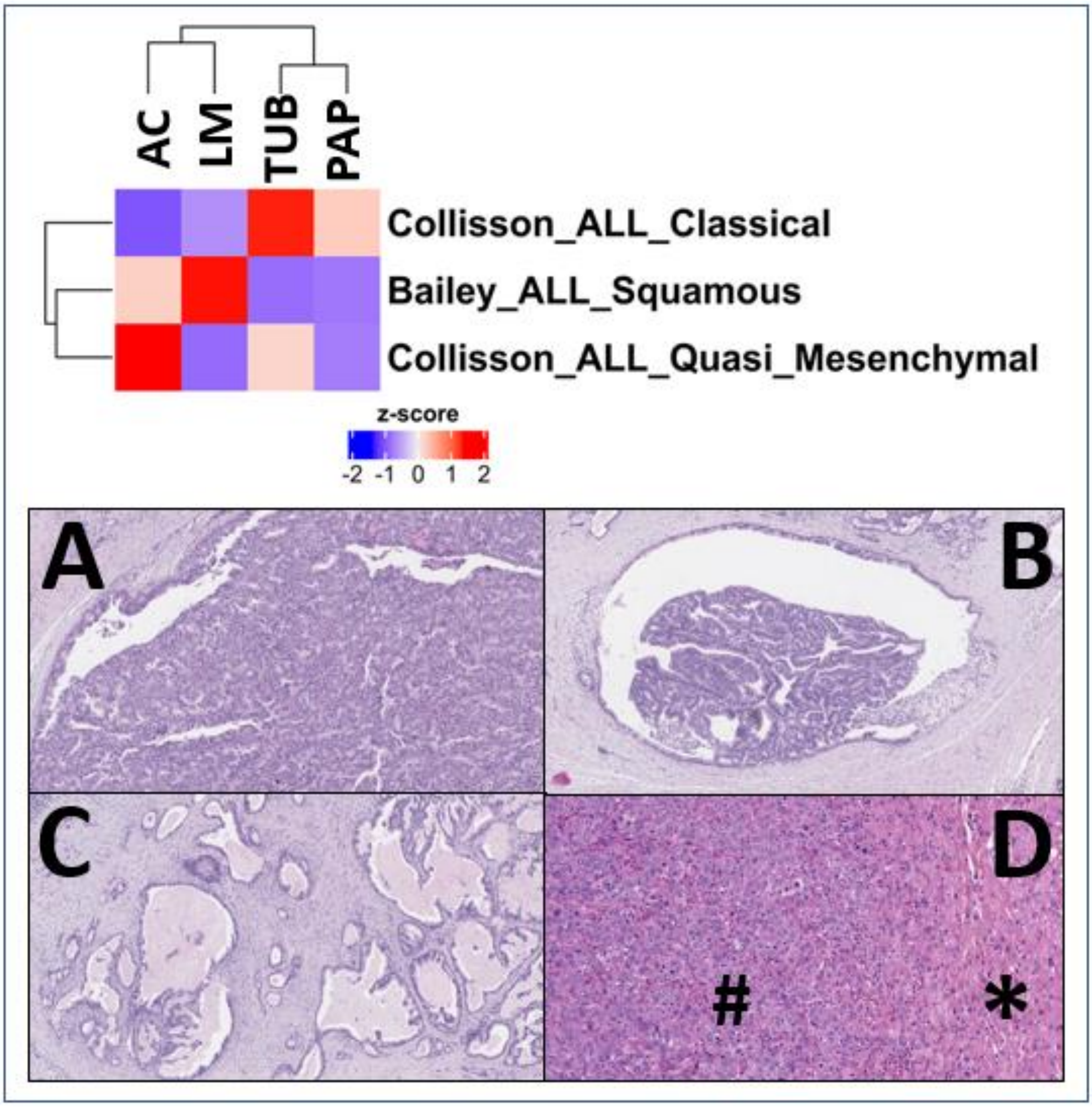

Supplement: Supplementary file 1 — Supplementary Material [file 41379_2022_1143_MOESM1_ESM.pdf]
